# Reviewing the evidence on health financing for effective coverage: do financial incentives work?

**DOI:** 10.1136/bmjgh-2022-009932

**Published:** 2022-09-20

**Authors:** Damien de Walque, Eeshani Kandpal

**Affiliations:** Development Research Group, World Bank, Washington, District of Columbia, USA

**Keywords:** Health economics, Maternal health, Review

## Abstract

The widening gap between improving healthcare coverage rates and stagnating health outcomes across low-income and middle-income countries highlights the need for investments in quality of care, in addition to access. New research, presented in a World Bank report, examines one type of relevant policy reform: performance-based financing (PBF), which is a package reform that always includes performance pay to front-line health workers and often also provides facility autonomy, transparency and community engagement. A large body of rigorous studies and new analysis show that in under-resourced, centralised health systems, PBF can result in gains to service utilisation, but only has limited impacts on quality. Even the relative benefits of PBF on service utilisation are less clear when compared with (1) direct facility financing which provides front-line facilities with operating budgets and provider autonomy, but not performance pay and (2) demand-side financial support for health services (ie, conditional cash transfers and vouchers). Thus, the central component of PBF—the performance pay—appears to add little value over flexible payment systems and provider autonomy. The analysis shows that this lack of impact is unsurprising because most of the constraints to improving quality do not lie with the health worker in these settings. While PBF was conceived as a complex package ‘blueprint’, we review the evidence to conclude that only some elements seem to make sense. To improve quality of care, health financing should pivot from performance pay while retaining the elements of direct facility financing, autonomy, transparency and community engagement.

**Summary box**

- There is little evidence of impacts supporting across-the-board performance pay in under-resourced, unfinanced health systems in low-income and middle-income countries.
- Direct facility financing with autonomy and accountability can deliver many gains at lower cost and with relatively easier implementation than performance pay interventions.
- Two-thirds of poor quality is not attributable to poor worker effort, but rather to structural quality and worker knowledge. In such settings of many competing constraints, including several that are systemic, performance pay is unlikely to be a silver bullet.
- Demand-side policies such as, but not limited to, conditional cash transfers and vouchers, can supplement supply-side interventions.
- Without addressing systemic constraints, such as the mode of governance, bureaucratic norms and the nature of public financial management, technocratic approaches are unlikely to radically overhaul health systems and achieve the Sustainable Development Goal of health for all.
- Before designing health financing reform, policy-makers should: (A) Assess coverage versus effective coverage to identify ‘low-hanging fruit’ for performance pay; (B) Assess constraints to quality of care to ensure they are in locus of control of the front-line worker and (C) Baseline utilisation should have room for improvement but not be so low as to indicate demand-side barriers.

## Introduction

**Figure 1:**
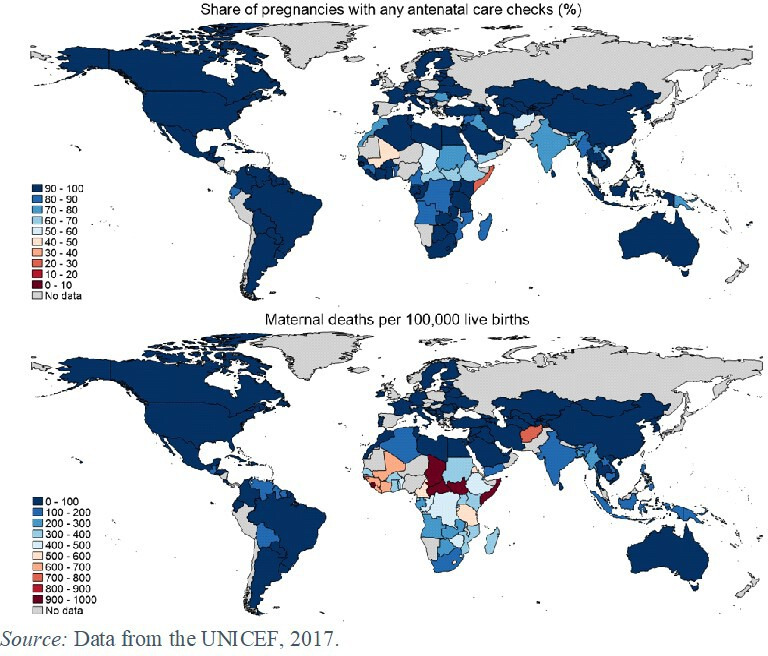
Antenatal care and maternal mortality rates around the world.

For many decades now, low-income countries have faced poor health outcomes despite steadily improving access to primary healthcare, suggesting that the care provided is often of inadequate quality. While reasons such as the social determinants of health may also contribute to this gap between access and outcomes,^1^ quality is often a binding constraint; for instance, figure 1 shows that most of the world now has access to antenatal care (ANC), but maternal deaths remain staggeringly high in many low-income countries. Indeed, most low-income and middle income country (LMIC) neonatal and maternal deaths are ‘amenable’, which is to say that they could be prevented by improving the quality of care.^2 3^

The COVID-19 pandemic has further threatened progress towards the goal of Universal Health Coverage,^4^ with ministries of health needing to find ways to return to prepandemic levels of service delivery while ensuring higher standards of care. Thus, sustained investments aimed at improving service quality—not just the quantity—are urgently needed if we are to achieve the Sustainable Development Goal of good health and well-being for all. The reform of health financing could help, perhaps by financing front-line facilities while pivoting away from input-based financing and towards performance-based financing (PBF). PBF projects are package reforms that include performance pay among other critical features, including public financial management (PFM) reform, health facility autonomy, decentralisation, supportive supervision for the front lines and community engagement. PBF was developed and scaled up on a body of evaluative empirical evidence,^5–7^ under a number of strong, often contradicting, theoretical assumptions on the centrality of financial incentives in health service delivery.^8 9^ In well-sourced, decentralised settings where provider effort is the central challenge to improving health, performance pay may have the scope to improve quality of care. We ask whether in contexts with systemic constraints, a lack of front-line autonomy, exacerbated by PFM issues, strong-but-corrosive bureaucratic norms, and other contextual aspects, performance pay can meaningfully improve service delivery.

A key player in health financing has been the World Bank’s Health Results Innovation Trust Fund (HRITF), which supported and evaluated LMIC governments in paying providers based on their results for the provision of maternal, newborn and child healthcare. At its peak, the PBF portfolio comprised 36 projects that spanned 28 countries in Africa, East Asia and Pacific, Europe and Central Asia, Latin America and the Caribbean, Middle East and North Africa, and Southeast Asia. These PBF projects represent more than US$2.5 billion in World Bank funding, with the majority being implemented in sub-Saharan Africa. As of June 2017, 32 of 46 sub-Saharan African countries had piloted or expanded PBF interventions.^10^

This large and rapid extension of PBF based almost entirely on external financing has been critiqued as having been a ‘donor-driven fad’ that did not draw on context-relevant evidence or expertise, while imposing a single model across the world without accounting for long-term consequences on diverse health systems.^11 12^ An important critique also questions the very assumptions supporting PBF pertaining to the salience of financial incentives as opposed to other means of supporting front-line service delivery. A response to this critique stresses that PBF catalysed a paradigmatic shift away from input-based financing and engendered systemwide improvements in transparency and accountability in their countries.^13^ This response also emphasises the role played by local stakeholders in adapting the basic design of PBF to local health systems, especially for scaleup—both by responding to local needs and integrating the underlying principles of sustainability, transparency and a results-link into the existing health system. A recent commentary in this journal points to this debate and calls for evaluations of PBF, particularly those that go beyond average effects and discuss heterogeneity within the approach.^14^ This paper aims to synthesise what we have learnt from the large HRITF-funded body of impact evaluations, and the necessary revisiting this evidence entails of the initial assumptions underpinning PBF. We review the evaluative evidence on the topic of PBF, discuss key highlights from a new World Bank report on health financing,^15^ and draw overarching messages for the design of health financing reform.

## Do financial incentives work?

A new report from the World Bank presents a comprehensive assessment of the evidence on PBF, drawing on harmonised data from rigorous impact evaluations of such health financing reforms. The report uses effective coverage as a measure of performance, which is a metric that combines simple healthcare coverage with minimum content and quality. Effective coverage and its two components, coverage and quality, can be represented on an ‘effective coverage contour’ with the measure of quality on the vertical axis and the measure of coverage on the horizontal axis. Each isocurve represents different combinations of quality and coverage that yield the same level of effective coverage. The closer an isocurve is to the upper right corner of the graph, the higher is the effective coverage rate for a condition.

**Figure 2:**
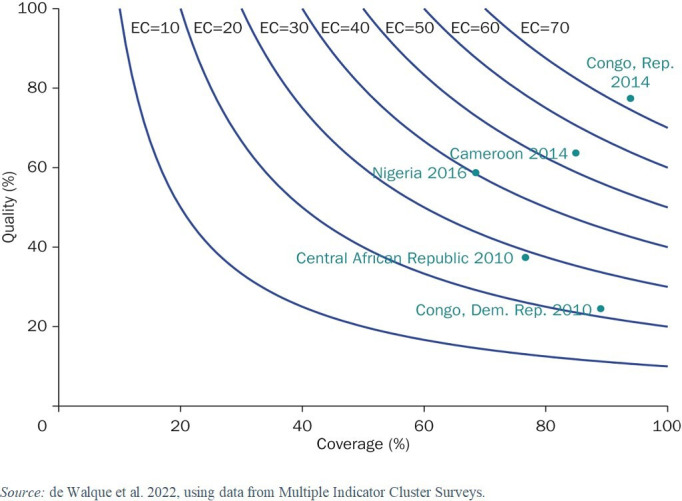
Effective antenatal care coverage in five sub-Saharan African countries. Note: Coverage: per cent of women giving birth who had 1+ ANC visits. Quality: of those with coverage, the per cent who had 4+ ANC visits with a skilled provider, blood pressure taken and blood and urine samples taken (correct treatment). ANC, antenatal care; EC, effective coverage.

Figure 2 illustrates with an example using ANC in five sub-Saharan countries: there is a consistent—and often large—gap between coverage and effective ANC in five representative sub-Saharan African countries, Cameroon, the Central African Republic, the Democratic Republic of Congo, Nigeria and the Republic of Congo. For the best performer, the Republic of Congo, coverage is near universal, but effective coverage is below 80%. For the worst performer, the Democratic Republic of Congo, the gap is 4.5-fold: approximately 90% of all women receive ANC, but only about 20% receive effective ANC.

The performance pay component of PBF centres on the idea that the quality of care is within the front-line facility or worker’s locus of control. While that may appear to be the case, how much of observed underperformance in fact is under the facility or worker’s control? Focusing on technical quality, we formalise our thinking using a formal decomposition of the causes of underperformance—shortfall relative to the international protocol—through a theoretical framework and an empirical application to ANC in the five sub-Saharan African countries presented above in figure 2. This framework decomposes three constraints to quality of care and describes the various levels at which they lie.^16^

First is the issue of inadequate technical capacity at the health facility, which may need to be addressed through centralised procurement and staffing. Indeed, the degree of centralisation within governance structures—of which procurement is but one example–itself is a complex one, and likely reflects political economy considerations and challenges that are well outside the locus of control of the front-line facility. In practice, this means that in centralised health systems (the institutional reality in many low-income countries), front-line health facilities may not have an operating budget that they control. So, if a piece of equipment breaks or there is a drug stockout, a health worker may not be able to provide necessary care because they lack the infrastructure. Addressing gaps in technical capacity requires giving front-line facilities a budget under their control and some say in staffing, at least to meet temporary spikes in demand—such as the ability to hire contractual staff during times of peak malaria transmission. Such an operating budget may be tied to facility-level performance targets but can equally be unconditional.

Second is the problem of inadequate health worker knowledge—workers may simply not know what services to provide. Medical knowledge is typically produced further up the health system in medical schools and controlled through national curricula and licensing requirements. Addressing gaps in worker knowledge would thus require a completely distinct set of interventions.

Finally, there is the issue of inadequate health worker effort and motivation and satisfaction, which may be reflected in the worker’s comportment or attitude. Such aspects are the only component of underperformance in this framework that might lie directly under the health worker’s control and thus would be the only component potentially addressable using performance pay to the worker. In other words, performance pay can incentivise front-line health facilities and workers, but only partially addresses constraints to quality. So, such a diagnostic assessment of the constraints ex-ante would tell us how much scope performance pay has to improve quality. Nonetheless, financial incentives are not the only or the most effective way of improving either. There are several sources of worker motivation and non-pecuniary approaches may at least as effective at motivating staff.^17^ Crucially, an extensive literature grapples with the overarching role of political economy and local bureaucratic norms that drive and mediate all technical approaches such as PBF.^18–20^ This literature suggests that technical approaches not integrated into the local political economic context and without addressing systemic constraints—as critics have argued PBF does not—will fail, particularly in terms of sweeping improvements.

An empirical application of this decomposition of the constraints to quality further illustrates this point. This application shows that despite years of investments in physical infrastructure and medical training, the five sub-Saharan African countries included in figure 2 continue to have pervasive gaps in physical infrastructure and health worker knowledge. ‘Know-can-do’ gaps—that is, care that the provider did not provide despite having all the necessary equipment and knowledge—explain less than one-third of all underperformance relative to WHO protocols for ANC. In other words, performance pay to the worker would—at best—only improve quality by a third. The remaining two-thirds of the constraints to quality do not lie with the health worker in any of the five contexts studied. This exercise thus illustrates the limits of performance pay in improving quality in under-resourced settings that highlights multiple competing constraints.

Perhaps unsurprisingly then, a consensus in the literature is that while PBF projects have led to gains in some aspects of primary health service delivery, some of the largest impacts observed are on structural quality, with small or even mixed impacts on process or clinical quality giving rise to questions of efficacy and effectiveness.^21^ These questions become even more salient when comparing PBF to simpler, less expensive approaches deploying financing to both the facility and the patients: direct facility financing (DFF) and conditional cash transfers (CCT) and vouchers to patients. While the literature identifies many other approaches to improving patient demand—such as citizen scorecards, knowledge interventions, even women’s empowerment programmes—we focus on financial incentives. This focus is driven by the idea of comparing financial incentives on the demand and supply sides. This, of course, does not imply that non-pecuniary approaches might be as or more effective than pecuniary ones, but their role is outside the scope of this analysis. DFF, an emerging policy counterfactual to PBF, shares many features of PBF projects in terms of operating budget to the front lines as well as autonomy over how to disburse that budget—but does not include performance pay.^22^

Several evaluations of PBF programmes have found that a comparison group that maintains resource neutrality and includes decentralisation can lead to equivalent gains in coverage although with differential impacts on quality of care.^23–25^ While PBF interventions provide an unconditional core budget and additional financial incentives conditional on performance, DFF interventions only provide additional unconditional financing. Often the financing is accompanied by autonomy, community engagement and supportive supervision. Furthermore, PBF and DFF are perhaps best viewed as mechanisms that leverage input financing and user fees.^26^ In PBF facilities, providers were actively encouraged to lower user fees or even waive out-of-pocket payments as a strategy to boost demand.

Pooled data from all five projects in the World Bank health financing portfolio that directly compared PBF and DFF sheds further light on the comparison of these two approaches. These five projects were implemented in Cameroon, Nigeria, Rwanda, Zambia and Zimbabwe. The pooled analysis shows that all these forms of financial incentives can increase health service utilisation, but except for increases in institutional deliveries, performance pay adds little gain over DFF. Furthermore, PBF can also be costly and difficult to implement—even relative to DFF, which in itself may have implementation challenges. While, at least in theory, performance pay makes sense in high-quality, decentralised health systems, its potential is limited in centralised, under-resourced health systems that have gaps at various points. DFF may be a more tractable first step in the latter settings.

The question of relative effectiveness of PBF also draws on a systematic review and meta-analysis of evidence from 52 programmes in 30 countries comparing the impacts on reproductive, maternal and child health service coverage across PBF, voucher and CCT programmes.^27^ All three financial incentives increase coverage of the considered indicators (modern family planning, institutional delivery, at least four ANC visits, at least one postnatal care visit, maternal tetanus vaccination and full child vaccination) but mean effect sizes are modest with little evidence of variation across intervention types. The only exception is ANC, where, in fact, CCTs significantly outperform PBF. This evidence, thus, suggests that cash transfers and vouchers can also be part of the solution in low-demand settings.

Finally, an in-depth examination of the salient criticisms of PBF interventions and key design elements presents several important lessons for sustainable health financing reform. For instance, in addition to the strategic incentivisation of priority services, a key difference between PBF and DFF interventions lies in the verification of facility performance reports. Under PBF, the quantity of services reported by the facility is verified at regular intervals—monthly or quarterly—by an external verification agency. Such verification requires sufficient local capacity to conduct the audits, as well as contributes substantially to project administrative costs^28^ and even creates incentives for collusion and gaming.^29–31^

Risk-based algorithms can reduce administrative costs and detect collusion in the data.^32^ However, because DFF interventions do not include strategic purchasing, they entirely avoid verification with its associated costs and implementation challenges. Of course, DFF projects also require that PFM be aligned with project design. For example, to receive and use operating budgets, health facilities must first be deemed as ‘spending units’ in the national charter of accounts, which is not always a straightforward process—although unlike verification, this issue needs to be addressed only once.

Another important criticism of PBF is that it generates unintended consequences,^33^ especially on the quality of service delivery^34^ and degrades health systems by crowding-out intrinsic motivation with extrinsic motivation. This criticism says that when the donor-financed PBF pilot ends and the source of the extrinsic motivation—the performance pay—is gone, all that remains is a work force that is now unmotivated and dissatisfied. While such intrinsic motivation crowding out has been examined both theoretically and empirically in the behavioural economics and social psychology literatures, the evidence comes from high-income settings that introduce payments to charitable and typically unincentivised tasks such as blood donation.^35–37^

LMIC evidence on this issue is at best inconclusive.^10^ Two studies that are often discussed are the PBF pilot in the Haut-Katanga province of DRC and the Malawian PBF pilot.^38 39^ In the DRC example, the authors report large decreases in worker motivation, but cannot disentangle the impacts of performance pay from those of an implementation error that led to an accidental 42% decline in the salary of workers in the PBF facilities. A large decline in salary, whether in the form of performance pay, may reasonably reduce worker motivation. Concerningly, a later study in the same context found persistent detriments to the health system as the salary decline led to greater staff attrition.^40^ In the Malawian pilot, authors report that PBF did not affect health workers’ intrinsic motivation levels. Thus, commentators have called for more field experiments on the incidence of intrinsic motivation crowding out in the context of performance pay.^41^

Further, paying workers for performance may have other impacts on their motivation, satisfaction, and well-being—beyond any crowding out of intrinsic motivation. To date, however, little rigorous evidence has examined the empirical foundations of the impacts of performance pay on worker motivation and satisfaction. A nascent body of literature considers these impacts and points toward contradictory findings from impact evaluations to call for more research on the influence of the context and design of PBF schemes.^42^

Rigorous quantitative evidence can be brought to bear on this question by pooling data from six studies conducted in Cameroon, the Kyrgyz Republic, Nigeria, Tajikistan, Zambia and Zimbabwe. The analysis leverages the randomisation of analytical units to the treatment to identify the impact of PBF versus business-as-usual on health worker motivation and satisfaction. By and large, it finds no evidence of significant impacts of performance pay—whether positive or detrimental—on any dimension of worker motivation, satisfaction or well-being. Thus, even though concerns regarding the potentially harmful effects of performance pay on worker motivation are theoretically grounded, there is at this point, little rigorous empirical evidence backing these concerns regarding worker motivation. There may be counterfactual or supplementary policies that outperform performance pay in terms of impacts on coverage or effective coverage but concerns about its impacts on human resources do not appear to be a reason to drop it from the policy toolkit.

## Conclusion

In summary, health facilities can deliver better results with certain elements common to PBF including budget autonomy, flexibility, transparency and unified payment systems. However, health facilities’ budgets can be impactful, and output-oriented if desired, even without the defining component of PBF—performance pay—especially in under-resourced settings. This finding highlights the centrality of considering and assessing systemic constraints before implementing narrower technical solutions. For instance, a nascent body of literature points to the importance of the link between PFM and health financing reforms.^43–45^ To this end, health financing reform design may benefit from starting with a diagnostics exercise that (1) identifies the constraints to both the provision of and access to high quality care, (2) benchmarks the local health system (eg, whether front-line facilities have operating budgets), (3) assesses the need to leverage demand and supply side policies and (4) identifies the appropriate sequencing of health financing interventions and necessary adjustments to PFM.

In emphasising the role of local conditions, including the nature of the health system and PFM, we join a growing body of work that emphasises the importance of context in designing effective and robust health systems.^46–48^ In conclusion, the measurement of healthcare quality and efficiency should be built into health systems reform, and local policy-makers and domestic constituencies should systematically ensure that the context and time are right for performance pay—that the constraints to quality lie within the front-line worker or facility’s locus of control, such that they can respond to performance pay by improving quality. If not, scarce resources will continue to be directed to inefficient uses.

## Data Availability

Data are available in a public, open access repository.
